# [Corrigendum] Bcl11b regulates enamel matrix protein expression and dental epithelial cell differentiation during rat tooth development

**DOI:** 10.3892/mmr.2026.14024

**Published:** 2026-09-16

**Authors:** Ziyue Li, Guoqing Chen, Yaling Yang, Weihua Guo, Weidong Tian

Mol Med Rep 15: 297–304, 2017; DOI: 10.3892/mmr.2016.6030

Following the publication of this paper, it was drawn to the Editor's attention by a concerned reader that, in the “*Protein extraction and western blotting*” subsection of the Materials and methods on p. 299, it was reported that 30 ng samples of protein were separated using SDS-PAGE, a concentration that would be three orders of magnitude lower than what is typical. Furthermore, regarding the “*Chromatin immunoprecipitation (ChIP) assay*” subsection of the Materials and methods on p. 298, the primer sequences described for targeting of the Msx2 promoter and the GAPDH control were identical to the sequences of the Msx2 and GAPDH primers reported in Table I, which were those used for RT-qPCR analysis of cDNA derived from reverse-transcribed mRNA. RT-qPCR primers are designed to target exon sequences in cDNA, whereas ChIP primers are designed to target genomic promoter regions; therefore, these primers would not have been expected to have shared identical sequences, given that physically distinct genomic regions were being targeted.

In their replies to these queries, the authors have confirmed that “30 ng” was a typo, and in the relevant subsection of the Materials and methods section, this should have been written as “30 μg”. Concerning the nature of the primer sequences, the authors confirmed that the same GAPDH primer was in fact used as a negative primer control for both the ChIP and the RT-qPCR analyses. However, the sequences used for the *Msx* gene for the RT-qPCR analysis, as correctly presented in Table I, were inadvertently copied across to the “*Chromatin immunoprecipitation (ChIP) assay*” subsection of the Materials and methods. In this section (p. 299, left-hand column, sentence commencing on line 4), the sequences of these primers should have been written as follows (the changed primer sequences are highlighted in **bold**): “Primers (forward **AGT GCT GCA GTT GGT CAT TG** and reverse **CCT GCA AAT AAC GGG GTT CA**) were used to amplify the Msx2 promoter”. The region from −1,200 to +100 bp relative to the Msx2 transcription start site (TSS) was selected as the sequence of interest. The RT-PCR primers for GAPDH were used as control primers.

In connection with the issue of presenting the correct sequences for the *Msx* gene for the RT-qPCR analysis, the authors have realized that Fig. 4B was labeled incorrectly. As described in the Materials and methods and the Results sections, the ChIP assay was performed to detect the Msx2 promoter region, rather than the Bcl11b promoter. The labeling for the lanes of the gel in Fig. 4B has therefore been corrected to reflect this, and the revised version of Fig. 4 is shown on the next page. In addition, certain corrections were required to the legend for this figure, and the specific changes that have been made are highlighted as follows in bold: “(B) PCR products of ChIP assay were verified by 2% agarose gel electrophoresis. Bcl11b, anti-Bcl11b antibody; input, cell lysate; Positive control, anti-RNA polymerase II antibody; GAPDH, GAPDH primer; **pMsx2, Msx2 promoter primer; Msx2, Msx2 primer.**”

The authors confirm that these errors did not affect the overall conclusions reported in this study. All the authors agree with the publication of this corrigendum, and the authors are grateful to the Editor of *Molecular Medicine Reports* for allowing them the opportunity to publish this. The authors regret that these errors were included in the paper, and apologize to the readership for any inconvenience caused; they also thank the reader for drawing these matters to their attention.

## Figures and Tables

**Figure 4.**
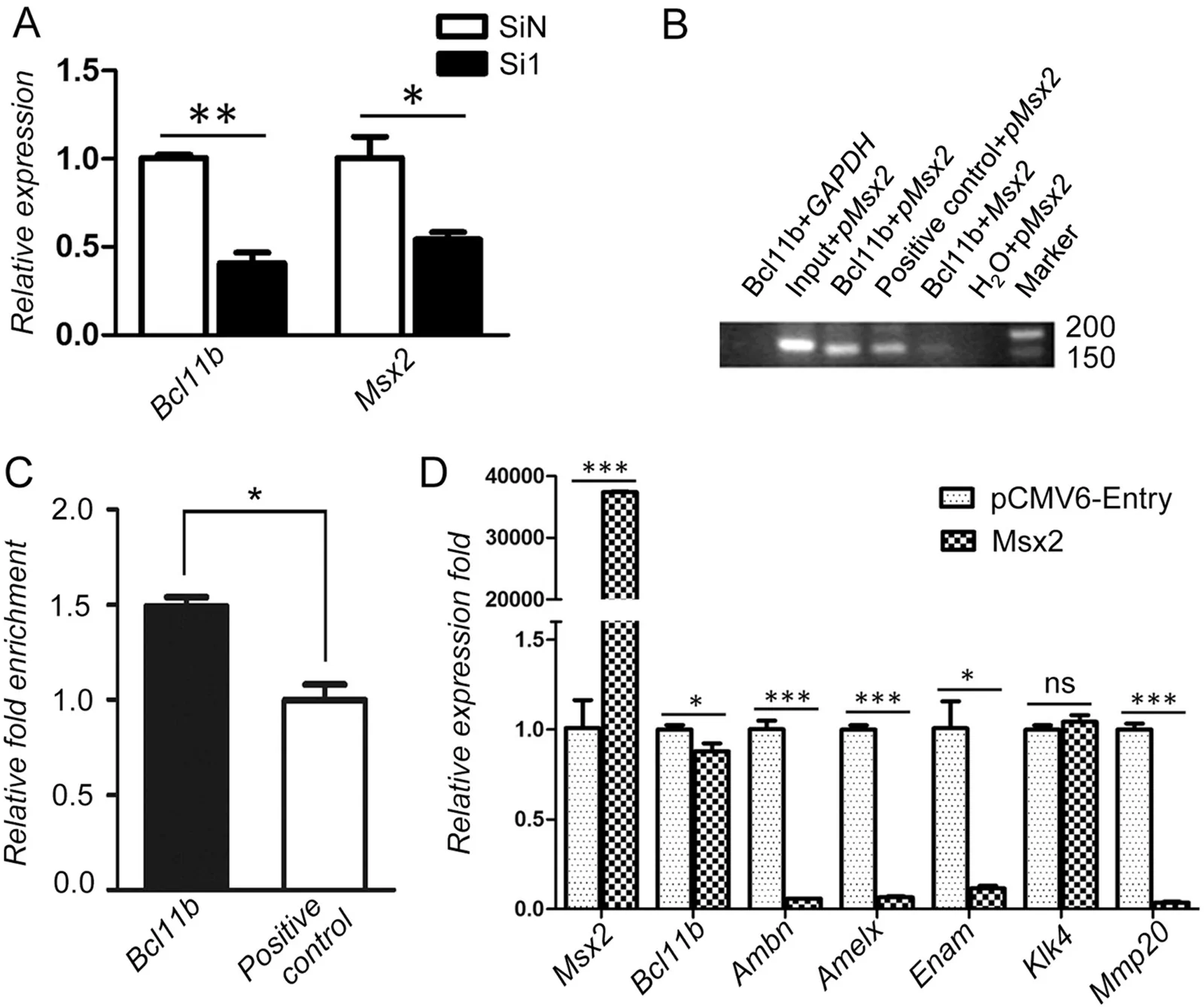
Msx2 regulates enamel-associated genes. Msx2 is a target gene of the transcription factor Bcl11b, as verified by ChIP assay. (A) Knockdown of Bcl11b led to a significant decrease in Msx2 expression. *P<0.05, **P<0.005, n=3. (B) (B) PCR products of ChIP assay were verified by 2% agarose gel electrophoresis. Bcl11b, anti-Bcl11b antibody; input, cell lysate; Positive control, anti-RNA polymerase II antibody; GAPDH, GAPDH primer; **pMsx2, Msx2 promoter primer; Msx2, Msx2 primer**. (C) Analysis of relative fold enrichment of ChIP. Quantitative PCR result between the Bcl11b experimental group and the positive control group. Student's t-test was used for statistical analysis. *P<0.05, n=3. (D) Relative expression of enamel-associated genes following Msx2 overexpression. *P<0.05, ***P<0.001, n=3. ns, not significant; Bcl11b, B-cell CLL/lymphoma 11B; Msx2, Msh homeobox 2; Amelx, amelogenin, X-linked; Ambn, ameloblastin; Enam, enamelin; Mmp20, matrix metallopeptidase 20; Klk4, kallikrein related peptidase 4; PCR, polymerase chain reaction.

